# Takotsubo Cardiomyopathy Associated with Levothyroxine Over-replacement

**DOI:** 10.17925/EE.2017.13.01.30

**Published:** 2017-04-03

**Authors:** Ana Margarida Balsa, Ana Raquel Ferreira, Márcia Alves, Joana Guimarães

**Affiliations:** 1. Department of Endocrinology, Diabetes and Nutrition, Hospital Centre of Baixo Vouga, Aveiro, Portugal; 2. Department of Cardiology, Hospital Centre of Baixo Vouga, Aveiro, Portugal

**Keywords:** Thyrotoxicosis, hyperthyroidism, Takotsubo cardiomyopathy, iatrogenic disease

## Abstract

Takotsubo cardiomyopathy (TC) is characterised by acute, transient left ventricular apical ballooning precipitated by emotional or physiologically stressful stimuli and has been previously associated with Grave’s disease based on a few clinical reports. More recently, the association with exogenous thyrotoxicosis and radioiodine-induced thyroiditis has also been described. Iatrogenic hyperthyroidism on patients on levothyroxine replacement therapy for hypothyroidism has not been reported as a cause of TC. The authors describe two female patients with TC associated with levothyroxine over-replacement. A 74-year-old and a 48-year-old female patient, medicated with levothyroxine (respectively, 2.27 μg/kg and 1.85 μg/kg) for autoimmune thyroiditis were admitted to our emergency room with precordial pain. The first had an electrocardiogram with ST-segment elevation in the anterior precordial leads, and the latter had sinus tachycardia with deep T-wave inversion and QT interval prolongation. Further investigation revealed a mild elevation of cardiac biomarker levels and severe apical hypokinesis, but no significant coronary lesions on catheterisation. The suppressed thyroid stimulating hormone (TSH) levels were verified in the cardiac intensive care unit: 0.21 and 0.07 mIU/l (0.35–5.50) respectively. Both patients showed improvement of the apical hypokinesis on the discharge echocardiogram and normalisation of cardiac biomarker levels. Levothyroxine dose was reduced. This case report focuses on the cardiovascular risks of thyrotoxicosis, emphasises the importance of correct dose adjustment on patients under levothyroxine replacement therapy and stresses that TSH should be determined in patients presenting with acute coronary syndrome and typical findings of TC.

Takotsubo cardiomyopathy (TC), frequently referred as stress-induced cardiopathy, apical ballooning syndrome or ‘broken heart’ syndrome, is characterised by left ventricular ballooning and transient systolic dysfunction. The Japanese term ‘takotsubo’ means a trap to catch an octopus, and its shape resembles this condition’s left ventricular systolic appearance.^1^

Clinically, TC can mimic an acute coronary syndrome (precordial pain, ST segment elevation or T-wave inversion in the electrocardiographic anterior precordial leads, elevation of cardiac biomarkers), but the segmental motility changes happen in the absence of coronary obstruction.^2^

TC is frequently associated with the occurrence of a stressful event, physical or emotional, however, the pathogenic mechanism is not fully understood. There is growing evidence of occurrence of TC in patients with thyrotoxicosis, pointing out that this may be a potential precipitating factor.^3^

Classically, TC has been mostly classified according to its ventricular involvement (uni- or biventricular), ballooning pattern (apical, basal, mid-ventricular and localised) and typical or atypical presentation.^4,5^ A new classification has been proposed regarding potential triggers, stating that secondary forms of TC (including patients with thyrotoxicosis) are associated with a worse longterm prognosis compared with primary forms.^6^

## Case report

### Case 1

A 74-year-old female patient with history of auto-immune thyroiditis, dyslipidemia and rheumatoid arthritis, medicated with levothyroxine (2.27 μg/kg), calcium carbonate and cholecalciferol 1,250 mg plus 400 IU id, simvastatin 20 mg id, escitalopram 10 mg id and macrogol 10,000 mg id. She presented in the emergency room with chest pain with sensation of tightness, nausea and vomiting, occurring after physical effort and lasting for six hours. She reported increased anxiety levels and fatigue during the past two weeks, but denied other significant clinical complaints such as weight loss, tremor, anxiety, sweating, increased appetite or increased bowel movements.

**Figure 1:**
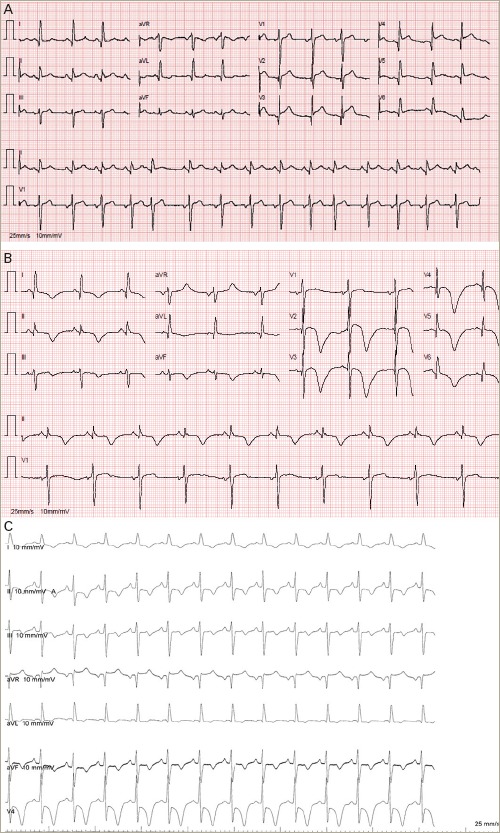
Electrocardiogram – patient 1 on admission (A) and two days following (B), showing T-wave invertion; patient 2 on admission (C)

**Figure 2:**
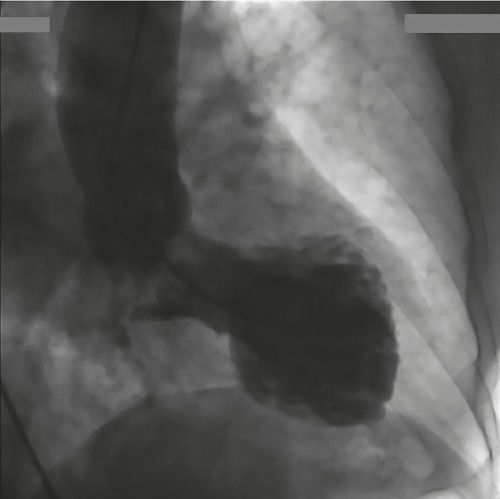
Cardiac catheterisation of the first patient, with a ventriculogram (A) showing the typical apical ballooning of Takotsubo cardiomyophathy

The electrocardiogram (ECG) showed ST-segment elevation in the anterior precordial leads (see *Figure 1*) and in the echocardiogram there was significant hypokinesis of the anterior and inferior portions of the apex with left ventricular systolic dysfunction (left ventricular ejection fraction [LVEF] 43%). Blood tests showed a rise of cardiac biomarkers: troponin value of 7.43 ng/ml (0.00–0.07). Cardiac catheterisation (see *Figure 2*) was performed and no evidence of coronary disease was found.

The suppression of TSH was detected in the cardiac intensive care unit: 0.21 mIU/l (0.35–5.50), with a free T4 of 1.43 ng/dl (0.80–1.80). Levothyroxine dose was then reduced. The patient showed great recovery, with rapid decrease of cardiac biomarkers and good clinical outcome. She was asymptomatic on discharge. The echocardiographic re-evaluation after three months showed complete reversion of apical hypokinesis.

### Case 2

A 47-year-old female patient with history of auto-immune thyroiditis, hypertension, dyslipidemia and non-Hodgkin lymphoma in complete remission, medicated with levothyroxine (1.85 μg/kg), acetylsalicylic acid 100 mg id, telmisartan/hydrochlorothiazide 40/12.5 mg id and pitavastatin 2 mg id.

She was admitted to the emergency room with palpitations and chest pain radiating to her left arm that started after emotional stress. She mentioned that she was experiencing effort-related retrosternal discomfort lately. She did not refer to other prior clinical complaints. The ECG showed sinus tachycardia (144 bpm), symmetrical T-wave inversion in the left ventricular leads, associated with prolonged QT interval (see *Figure 1*). The subsequent complementary workup showed a discrete rise in cardiac biomarkers (troponin levels of 0.28 ng/ml, 0.00–0.07) and apical hypokinesis without significant coronary obstruction on coronary angiography. She also had a TSH level inferior to the lower limit of the reference range: 0.07 mIU/l (0.35–5.50).

Similarly to what was previously described, her levothyroxine dose was adjusted and there was normalisation of cardiac biomarkers during inpatient care (troponin level of 0.09 ng/ml, 0.00–0.07 on the fourth day after admission).

Cardiac magnetic resonance was performed three weeks after discharge, it did not show any signs of late myocardial enhancement and already showed improvement of the morphological abnormalities previously described. Her echocardiographic re-evaluation after three months showed normal left ventricular contractile function.

## Comment

Many clinical manifestations of thyrotoxicosis are due to thyroid hormone action on the myocardial tissue, peripheral circulation and sympathetic nervous system, leading to a hyperdynamic state. Thyroid hormones contribute, directly and indirectly, to an increase of myocardial contractility and heart rate, thus increasing cardiac output, myocardial oxygen consumption, systolic blood pressure and pulmonary pressure, while there is a decrease in peripheral vascular resistance and in diastolic pressure.^7^ The main cardiovascular complications of thyrotoxicosis are atrial fibrillation, heart failure and pulmonary hypertension.^8^ Thyrotoxicosis is also associated with an increased risk of angina and even acute coronary syndrome, which is thought to be explained by the increase of myocardial oxygen demand caused by thyroid hormones, potentiating the occurrence of ischaemic events.^9^ Therefore, it is important to include TSH evaluation in the initial assessment of patients with those conditions.

Several reports support the association between TC and thyrotoxicosis. Although the mechanism is not fully understood, it is generally considered to involve an abnormal myocardial response to the circulating levels of catecholamines.^10^ The fact that there is an increased sensibility to catecholamines in thyrotoxicosis would justify its potential association with TC. This increased sensibility may entail an over-expression of G protein, down-regulation of the catalytic subunit of cardiac-specific adenylyl cyclase or an increased density of beta-adrenergic receptors. These receptors are found more densely spaced in the ventricular apex, which substantiates this segment’s vulnerability to hypokinesis in TC.^11^

There is no specific treatment for TC. This condition is managed with supportive therapy and usually results in rapid resolution of symptoms and reversion of the left ventricular dysfunction.^12^ It is recommended to solve potential underlying factors, when those can be identified: emotional or physical stress reduction and, as in the present cases, approach to thyrotoxicosis.

Both our patients presented with precordial pain, there was no strong clinical suspicion of pheochromocytoma or cerebrovascular disease and coronary artery disease was excluded. TC diagnosis was established in both and on the first case no drug or other precipitating factor was found other than excessive supplementation with levothyroxine, despite subclinical levels. The second patient referred to emotional stress, adding to the excessive supplementation with levothyroxine.

These case reports alert for the cardiovascular risks of sustained thyrotoxicosis, stressing the relevance of adequate dose-adjustment and regular clinical monitoring of patients on thyroid hormone replacement therapy, especially those with other cardiovascular risk factors.
